# Sensory and Motor Difficulties in Autism

**DOI:** 10.1192/j.eurpsy.2022.208

**Published:** 2022-09-01

**Authors:** T. Gargot

**Affiliations:** Hôpital Bretonneau, Child And Adolescent Psychiatry, Tours, France

## Abstract

Autism Spectrum Disorder (ASD) is a neurodevelopmental disorder characterized by difficulties in social interaction and communication, and by restricted and repetitive behaviors. A meta-analysis describes motor difficulties in ASD (Downey and Rapport, 2012) in 85% to 90% of cases (Liu et al. 2010). Electronic devices will help to better characterize these movement impairments (Gargot et al., in press). Another meta-analysis shows difficulties in sensorial integration in ASD, with a prevalence ranging from 45 to 95% (Ben Sasson, 2009). Sensorimotor contigencies are learned interactively (Jacquey et al., 2020), in a perception-action loop, an early milestone in development (Piaget, 1937). A cascade model (Bonnet-Brilhault, 2017) hypothesizes that social difficulties stem in sensorimotor difficulties (Cook, 2016; Neal, 2011; Dziuk et al. 2007; Kojovic, 2019), themselves atypically developed due to a peculiar neurobiological background. This model predicts that early sensorimotor reeducation could prevent the development of social difficulties, whereas the rehabilitation of social skills would improve only part of the impairment, that is driven by sensorimotor processes. How to target these issues? Grandin, 1992 and Edelson et al., 1999 validated the efficacy of deep pressure therapy in ASD. It is important to improve attractivity and decrease the stigmatization of this method. Oto is a compressive armchair with inflatable cushions controlled electronically. It records level of pressure and its duration, chosen by the user. A systematic review showed the efficacy of virtual reality in ASD (Mesa-Gresa et al, 2018). A CAVE device could habituate children to ecological audio-visual stimulations.

**Disclosure:**

Thomas Gargot was paid by the French ministry of research (Doctoral school), French ministry of health (médaille d’argent, CCA-AHU) for a PhD and CCA position. During his PhD, he prepared several scientific presentations with Dr Asselborn. This work led

